# Supplementary material for: Cyfip1 haploinsufficient rats show white matter changes, myelin thinning, abnormal oligodendrocytes and behavioural inflexibility
Source: Nat Commun. 2019 Aug 1;10:3455. doi: 10.1038/s41467-019-11119-7 (PMC6671959; doi:10.1038/s41467-019-11119-7)
Supplement: Supplementary file 1 — Supplementary Information [file 41467_2019_11119_MOESM1_ESM.pdf]

Cyfip1 haploinsufficient rats show white matter changes, myelin thinning, abnormal oligodendrocytes and behavioural inflexibility

Silva et al.

Supplementary Information

Pages 2 to 6: Supplementary Figures and Tables

Pages 7 to 19: Supplementary Methods

Supplementary Figures and Tables

Supplementary Figure 1 - Mean white matter skeleton mask in green superimposed on the mean fractional anisotropy (FA) template image from all the 24 rats used for Tract-Based Spatial Statistics. An optimal threshold of 0.2 was applied to the mean FA skeleton to create the binary white matter skeleton mask.

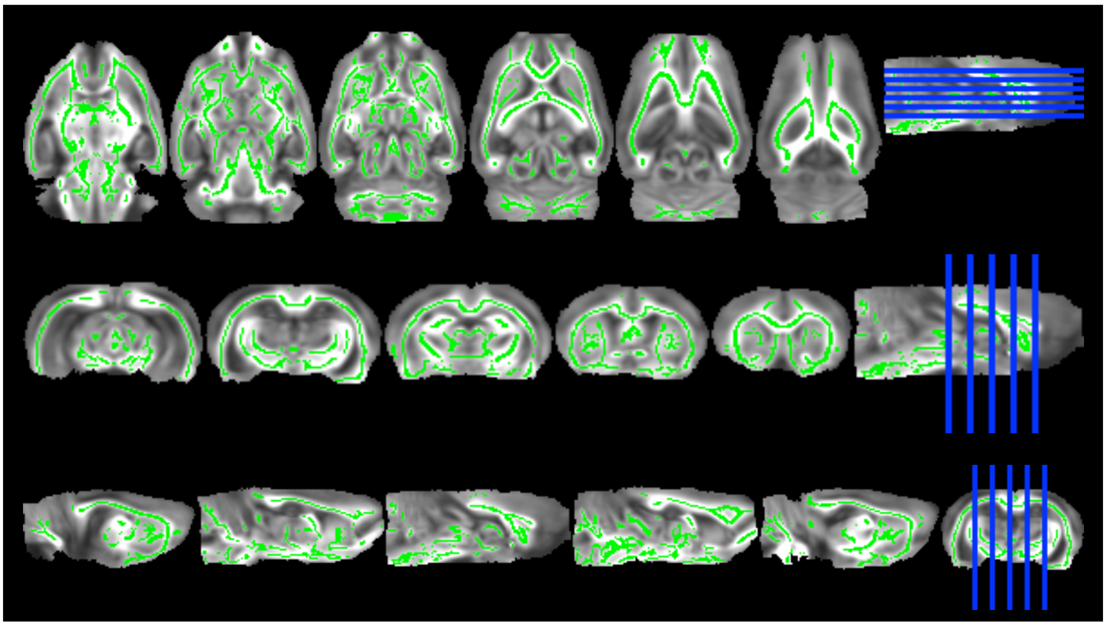

Supplementary Figure 2 - White matter binary masks manually delineated (using FMRIB software library) for the white matter tracts that showed significant differences in the Tract-Based Spatial Statistics analysis. The binary masks are represented in blue and are superimposed on the white matter skeleton in green.

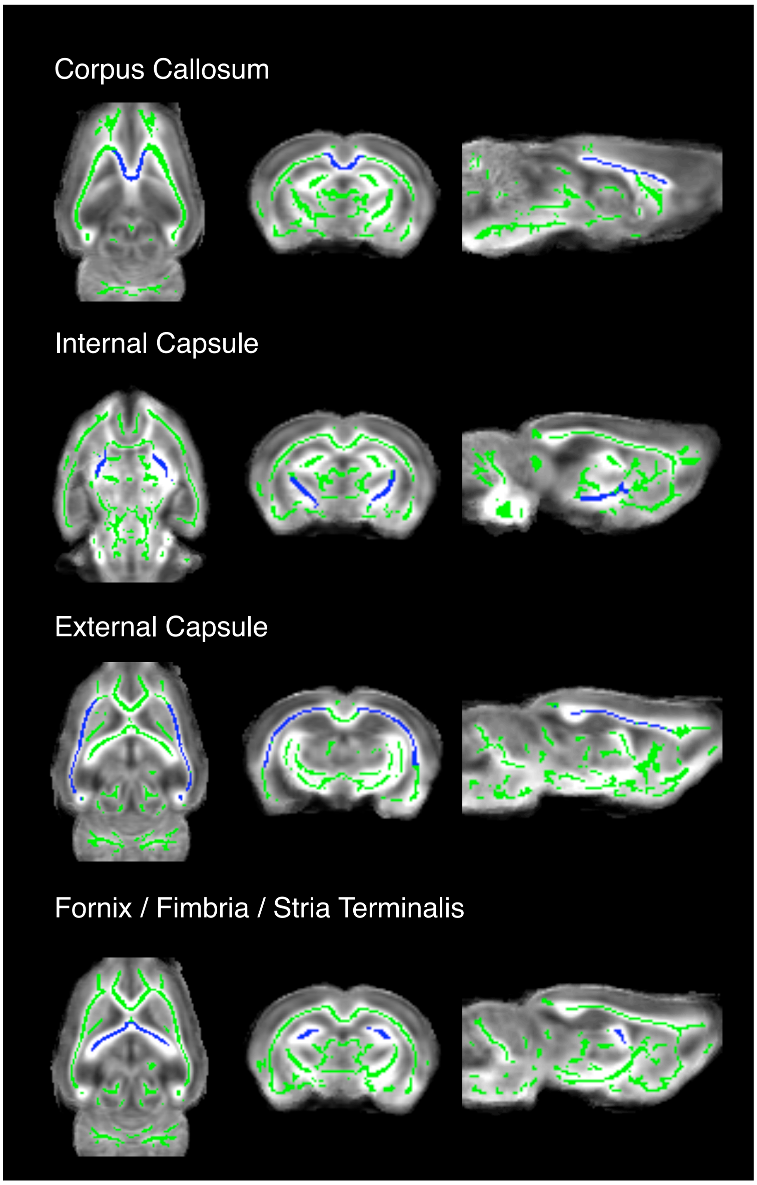

Supplementary Figure 3 – Relationship between g-ratio and axon diameter. a Mean g-ratio for each animal against mean axon diameters (wild-type n=5, *Cyfip1*$^{+/-}$ n=4), showing a high variability on axonal caliber within each group and **b** scatter plot of g-ratio values across all axon diameters wild-type (n=7145 axons) and *Cyfip1*$^{+/-}$ (n=5979 axons). Source data are provided as a Source Data file. WT = wid-type.

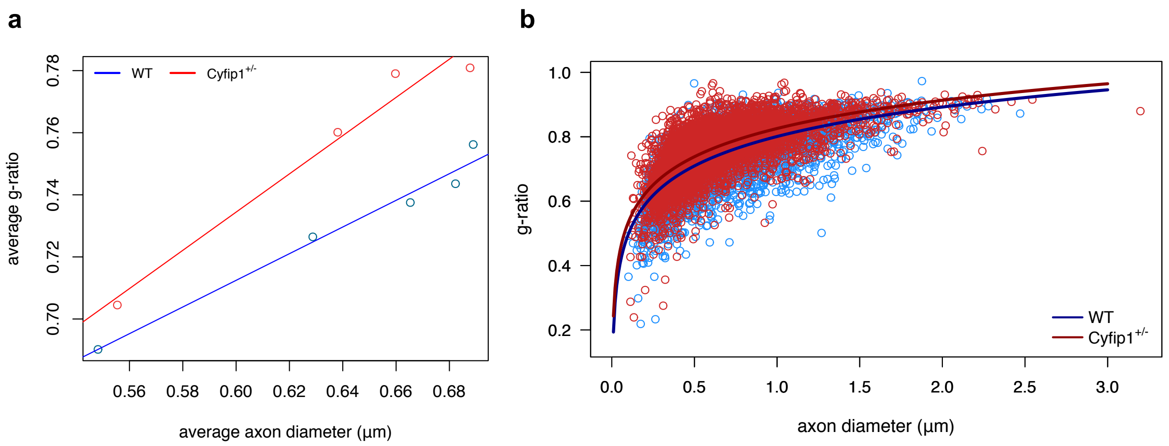

Supplementary Figure 4 - Flowchart overview of the touchscreen visual discrimination and reversal learning task: Following trial initiation, a stimuli pair (S+, S−) was presented on the screen on the left or right of the screen in pseudo-random fashion. Correct responses (a nose poke to the S+) were rewarded with 10% sucrose delivered into a recessed magazine on the opposite wall to the touchscreen. Incorrect responses (touching the S−) resulted in a time out (5s). Following an additional 5 second inter-trial-interval (ITI) a new trial began. The rats were trained until they reached criterion (80% correct on two consecutive sessions) following which the contingencies were reversed (S+ →S- and S- →S+).

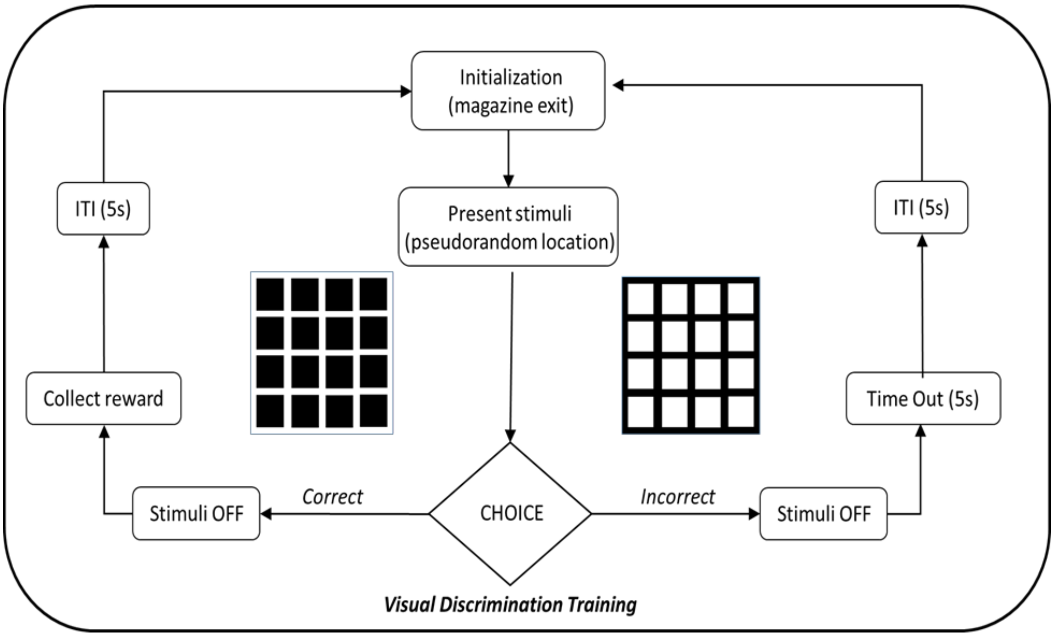

Supplementary Table 1 - a Numbers of rats completing each stage of the reversal learning task, and **b** the mean number of sessions and trials (in brackets) that it took to complete (i.e. reach criterion) across the different stages of the reversal learning task in both wild-type and *Cyfip1*$^{+/-}$ rats. Early Reversal was to a criterion of 50% correct, and Late reversal was classed as reaching performance of 80%, correct across two consecutive sessions in which completed <50 trials.

a

	**wild-type**	***Cyfip1***$^{+/-}$
**Magazine Training**	7	10
**Touch Training**	7	10
**Visual Discrimination**	7	9
**Early Reversal**	6	6
**Late Reversal**	4	5

b

	**wild-type**	***Cyfip1***$^{+/-}$
**Magazine Training**	**2.7** *(249)*	**4** *(325)*
**Touch Training**	**15** *(1041)*	**13** *(709)*
**Visual Discrimination**	**24.9** *(1031)*	**23.7** *(1082)*
**Early Reversal**	**14.1** *(322)*	**12.1** *(379)*
**Late Reversal**	**13** *(759)*	**10.6** *(815)*

Supplementary Methods

1. CRISPR-Cas9 targeting

Proprietary bioinformatics software (Horizon Discovery, St. Louis, USA) was used to design a short guide RNA (sgRNA) targeting a Protospacer Adjacent Motif (PAM) sequence within exon 7 of the rat *Cyfip1* gene (GGCAGATCCACAATCCATCCagg) on chromosome 1 (Supplementary Figure 5).

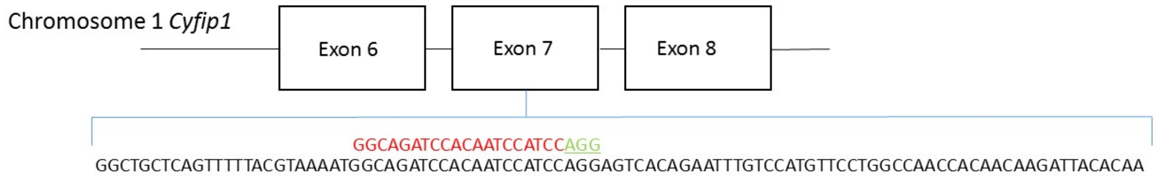

Supplementary Figure 5 - Targeting of the *Cyfip1* gene with sgRNA in exon 7. Targeting of the sgRNA was restricted to the first two thirds of the Open Reading Frame of the *Cyfip1* rat gene (first 21 of 32 exons, Refseq: NC_005100.4, NM_001107517.1). Upstream targeting at the 5'end was also avoided due to the possible presence of cryptic promoters. The guide was designed and selected with the use of a commercially available online CRISPR design tool (commercial algorithm) to locate the appropriate PAM sequences and ideal locations for the sgRNA. An oligo was generated containing the specific guide sequence along with the appropriate scaffold and the oligo then acted as a template in an in vitro transfection (IVT) reaction to generate the sgRNA.

An initial *in-vitro* assessment of efficiency of the sgRNA-Cas9 was performed by nucleofecting the sgRNA-Cas9 into rat C6 glial cells. Genomic DNA (gDNA) PCR products were subsequently generated from nucleofected C6 cells using primers flanking the sgRNA site (FOR: GCCAAAGCTTCCCCTAAAGT; REV: TGGGCGTCAAGTACATTCTG; 497bp amplicon). gDNA PCR products were screened for NHEJ activity and deletion mutations using

the SURVEYOR Cel-1 Mutation Detection Assay (Integrated DNA Technologies, following manufacturer's instructions, Supplementary Figure 6)

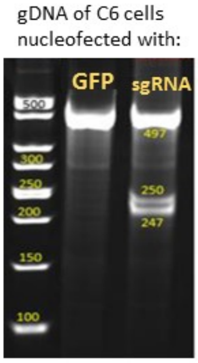

Supplementary Figure 6 – In-vitro assessment of targeting efficiency. gDNA was obtained from C6 glial cells nucleofected with sgRNA-Cas9 and Green Fluorescent Protein (GFP). C6 cells nucleofected with GFP only acted as a visual positive of successful nucleofection (cells fluoresce green) and a genetic negative control of NHEJ activity. Meanwhile, NHEJ activity is observed in C6 cells nucleofected with sgRNA-Cas9 via the observation of smaller cel-1 bands and the relative intensity of these bands compared to the intensity of the larger wild-type band (497 bp) can be used to estimate NHEJ

2. Embryonic microinjection of sgRNA-Cas9

Embryo donor female Long Evans rats were super-ovulated with pregnant mare serum (PMS) and given human chorionic gonadotrophin (HCG) 48 hrs post PMS administration. Females were immediately mated to stud males after HCG administration. Embryo donor females were euthanized 18-24 hrs after mating and their one-cell fertilized embryos were isolated by harvesting the reproductive tract and rupturing the ampulae. A total of 270 embryos were then put in culture media in a CO_2 incubator until ready for microinjection. All 270 one-cell stage embryos were microinjected with the validated sgRNA-Cas9 over four sessions and then implanted into synchronized pseudopregnant Long Evans recipient females, resulting in 18 live births.

3. Positive founders and confirmation of 4bp deletion

Of the 18 live births, 7 pups demonstrated NHEJ activity (39% efficiency) as measured by the SURVEYOR assay (with gDNA derived from P14 tissue biopsies). To further identify positive founders, sequencing experiments of the gDNA PCR products were performed and revealed one positive female showing a 4bp out of frame heterozygous deletion in exon 7 of the *Cyfip1*

gene at location Chromosome 1: 36974-36977 and a resulting bioinformatics prediction of an early stop codon in exon 8 (Supplementary Figure 7) Therefore, the efficiency ratio of generating rats carrying the required deletion was 5.5% (1 from 18 live births).

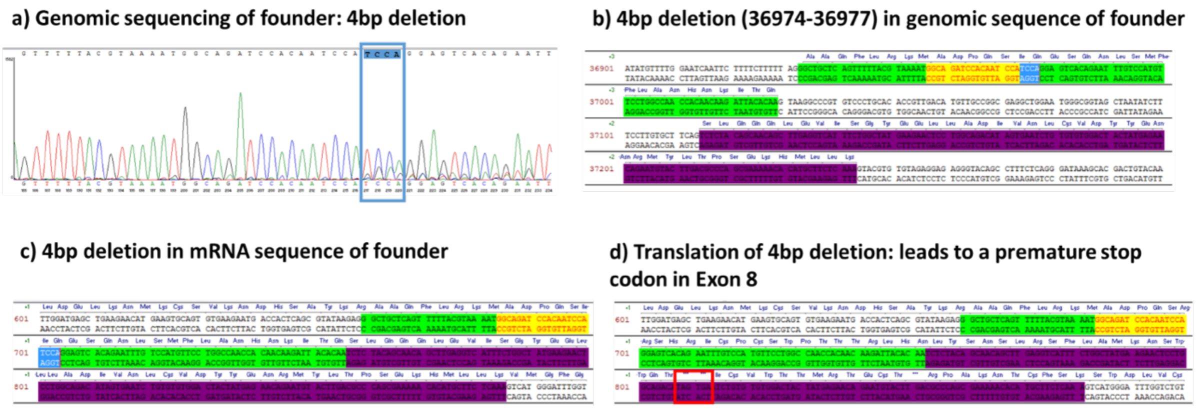

Supplementary Figure 7 - Genomic sequencing of the *Cyfip1* 4bp heterozygous deletion founder. a) Manual reading of each double peak in the sequencing chromatograph (ABI Sequence Scanner) show that upstream of the deletion, sequences from the wild-type and modified allele are identical. At the site of the deletion i) the sequence read becomes mixed and ii) the sequence of the secondary peaks from the modified allele align with wild-type sequence, except they occur 4bp further upstream. This reveals the size and position of the modified allele. **b)** The 4bp deletion in the founder genomic sequence is highlighted in blue (Exon 7 highlighted in green; sgRNA site highlighted in yellow; exon 8 highlighted in purple) **c)** 4bp deletion in the mRNA sequence of the founder. **d)** The translation of mRNA from the 4bp-deleted sequence is bioinformatically predicted to create an early stop codon in Exon 8 (codons TAG and TGA, region outlined by red box).

4. Assessment of potential off-target effects

Recent analysis has shown that Cas9-mediated off-target effects are relatively common[1], which found that 23% of 81-genome-editing projects exhibited OTEs across mouse and rat models. Anderson et al., 2018[1] highlights the importance of validating the specificity of CRISPR-Cas9 gene editing and recommends an assessment of sgRNA design, in-silico prediction of potential OT sites and importantly biological assays, that together allow confidence that any OT

engineered gene changes fall well below the background mutation frequency that can occur spontaneously.

To address this issue, we present an expanded analysis of potential OT sites in Supplementary Table 2. Here it is shown that the sgRNA guide designed against rat *Cyfip1* has a MIT specificity score[2] of 100% against the *Cyfip1* gene and an overall score of 73.3% when factored against 49 OT sites. Previously in authoritative reviews it has been considered that a cut-off score of 66%, and above is sufficient to effectively eliminate the likelihood of OTE[2] and this proposition has recently been confirmed by others[1]. Further, it has previously been shown that 2 mismatches (MM) – in concatenated or interspaced form - reduce Cas9 cleavage activity to low levels and can be further reduced to negligible levels if they occur within close proximity (12bp) to the PAM region[2]. Moreover, 3 concatenated mismatches were sufficient to eliminate detectable cleavage in the majority of loci tested, and more so, if these were interspaced and/or proximal to PAM. We also generated a list of top 10 potential off-target (OT) sites, based on the sgRNA sequence used (GGCAGATCCACAATCCATCCagg), and ranked using the MIT website http://crispr.mit.edu/. Closer inspection of our list of Top 10 OT sites in Supplementary Table 3, shows that all have a minimum of 3 MMs and sites 1 and 3 both possess a MM in close proximity to the PAM.

Supplementary Table 2 - MIT predicted off target sites for de Cyfip1 sgRNA in-silico.

	MIT specificty scores - using Benchling (previously MIT website)				Overall specificity score 73.3 %
Numbered Ots	Sequence	PAM	Score	Gene	Locus
	GGCAGATCCACAATCCATCC	AGG	100	Cyfip1 (ENSRNOG00000011945)	chr1:+114295724
OT1	GGAAAATCCACCATCCATCC	TGG	1.4		chr2:-95793954
OT2	GCCAAATCCACAATCCATGC	CAG	1.3		chr14:+5813530
OT3	GTATCATCCACAATCCATCC	AAG	1.3		chr4:+109037870
OT4	CTCAGAAACACAATCCATCC	CAG	1		chr3:+90820151
OT5	GGTAGCCCCACAATCCATCC	CAG	1		chr8:+90295695
OT6	TGTACAGCCACAATCCATCC	AAG	1		chr8:+45237516
OT7	GTCCGAACCCCAATCCATCC	TAG	1		chr2:-85528916
OT8	TGGAAATCCACAATCCATCT	CAG	0.9		chr2:+223864733
OT9	AGCAGATCCAGATTCCATCC	AGG	0.7		chr4:+72665650
OT10	AGCATATACACAATCCATTC	CAG	0.7		chr7:-113365387
OT11	TGCATACCCAGAATCCATCC	TAG	0.6		chr6:-128916162
OT12	AGCATAACCAGAATCCATCC	GGG	0.6		chr11:+79708047
OT13	GCTAGATCCTCAGTCCATCC	TGG	0.6		chr2:-172781048
OT14	AGCAGATGCCCAATCCATTC	GAG	0.6		chr18:-63836841
OT15	GACAAAGCCAAAATCCATCC	AGG	0.6		chr3:+28891768
OT16	GTCAGAGACAGAATCCATCC	AAG	0.6		chr4:-170865117
OT17	TGCACAGCCACAATCCTTCC	AGG	0.5		chr14:+13356994
OT18	AGCACTTCCACCATCCATCC	AGG	0.5		chr4:-180525716
OT19	AACTGATCCACAATTCATCC	CAG	0.5		chr8:-8347020
OT20	CACAGATCTACAATCCCTCC	CAG	0.5		chr3:-176292068
OT21	GGCAGATCCAGAACCCATCC	CGG	0.5		chr6:-43384274
OT22	TGTAGATCCTCAATACATCC	AGG	0.5		chr7:+67511010
OT23	TCCAGATCCAGAATCCCTCC	TAG	0.4		chr11:-36121578
OT24	GGCAGGTTCTGAATCCATCC	CAG	0.4		chr8:+24534839
OT25	GACACACCCACACTCCATCC	AGG	0.4		chr19:-55314147
OT26	AGCAGAGTCACAATCCATGC	AAG	0.4		chr2:-261967831
OT27	GGCAGCTGCGCAATCCATCA	TGG	0.4		chr3:-173954380
OT28	GGCAAATTCATGATCCATCC	AAG	0.4		chr10:+8354081
OT29	GGCACATTGACAATCCTTCC	CAG	0.4		chr2:-126822158
OT30	GGCGGACCCCCAGTCCATCC	AGG	0.4	Cyfip2 (ENSRNOG00000006557)	chr10:-31364956
OT31	TGCACATCCGCAATCCACCC	CAG	0.4		chr10:-92422723
OT32	TGCAGATACATATTCCATCC	CAG	0.4		chr15:+1789082
OT33	GGAATGTCCACAGTCCATCC	AGG	0.4		chr18:-31761206
OT34	GGAATTTCCACATTCCATCC	TGG	0.4		chr15:-61472675
OT35	AGCACATCCACTCTCCATCC	CAG	0.3		chr19:-30859164
OT36	GATAGATCCACTGTCCATCC	TGG	0.3		chr5:+63159170
OT37	AGCAGACTCACAATTCATCC	TAG	0.3		chr2:-85469475
OT38	ACCAGATCCACACTCCTTCC	CAG	0.3		chr5:+135998305
OT39	GGCACATCCACAATCCAGCA	AAG	0.3		chr1:+146539887
OT40	TTGAGATCCACAATCTATCC	TAG	0.3		chr2:+103959476
OT41	TGCAGAACCACTATCCATCT	CGG	0.3		chrX:-63694162
OT42	AGCAGCTCCAGAATCCATCA	TGG	0.3		chr1:-146566385
OT43	TGCAGATCTAAAATCCATCT	GGG	0.3		chrX:-143494363
OT44	GCCAGCTCCAAAATCCATCT	AAG	0.3		chr3:+127954078
OT45	TGCAGTTCCAGGATCCATCC	AAG	0.3	ENSRNOG00000020103	chr18:+30285706
OT46	GTCAGAACCAGAATCCCTCC	TGG	0.3		chr5:+75274315
OT47	GAAAGATCCATAATTCATCC	TGG	0.3		chr2:+253045233
OT48	TGCAAATCCACAGTCCATTC	AGG	0.3		chr5:-85205106
OT49	GCCAGGGCCACAATCCATAC	AGG	0.3	Grm3 (ENSRNOG00000005519)	chr4:-21535579

Supplementary Table 3 – Top 10 predicted off target sites for the Cyfip1 sgRNA with a breakdown of mismatches and individual MIT scores.

	Sequence	Genomic Coordinates	Mismatches	MM proximal to PAM (12bp core)	Interspaced MM	MIT score
**Injected US sgRNA sequence**	GGCAGATCCACAATCCATCCAGG	Chr1:+114295729	0	0	0	100
OT Site 1	GGAAAATCCACCATCCATCCTGG	chr2:-115534496	3	1	3	1.4
OT Site 2	GTATCATCCACAATCCATCCAAG	chr4:+173741171	4	0	0	1.3
OT Site 3	GCCAAATCCACAATCCATGCCAG	chr14:+5796700	3	1	3	1.3
OT Site 4	CTCAGAAACACAATCCATCCCAG	chr3:+97482256	4	0	2	1
OT Site 5	TGTACAGCCACAATCCATCCAAG	chr8:+43715985	4	0	4	1
OT Site 6	GTCCGAACCCCAATCCATCCTAG	chr2:-105200718	4	1	4	1
OT Site 7	TGGAAATCCACAATCCATCTCAG	chr2:+241911684	4	1	4	0.9
OT Site 8	AGCAGATCCAGATTCCATCCAGG	chr4:+137367617	3	2	3	0.7
OT Site 9	TGCATACCCAGAATCCATCCTAG	chr6:-138113252	4	1	4	0.6
OT Site10	AGCATAACCAGAATCCATCCGGG	chr11:-81022173	4	1	4	0.6

It is also important to notice recent discussions in the gene editing field as to the extent to which in-silico predicted OT sites (MIT website,Benchling) reflect true biological OT sites of Cas9-mediated activity. To some extent, this has been addressed experimentally by Anderson et al., 2018[1], whereby 30 robust OT sites were identified by two sequencing methodologies (whole-genome sequencing and TEG-sequencing) and found that 25 of these, i.e. 83%, where predicted using in silico methodologies. Therefore, in light of these results we can be reasonably confident that the Top 10 OTE table generated for the Cyfip1 sgRNA is reflective of bone-fide biological OT sites.

Furthermore, using a Surveyor assay, all the Top 10 predicted OT sites were negative, , i.e. no OTE were detected when we explicitly tested for them using wet methods. Corresponding gDNA PCR primer pairs were designed to flank each potential OT site (Supplementary Figure 8a). Extracted gDNA from the founder animal and wild-type controls were used as template for PCR, before each gDNA PCR product underwent the SURVEYOR assay (Supplementary Figure8b). The lack of NHEJ activity, as indicated by a lack of smaller secondary products, revealed no evidence for genomic disruptions at the selected sites and was further confirmed by sequencing the 10 OT site gDNA products (data not shown).

a) Top 10 potential off-target sites (MIT) for sgRNA

	Off Target Sequence	genomic coordinates	F primer	R primer
Injected gRNA sequence	GGCAGATCCACAATCCATCC**AGG**	chr1: 115302084-115302106		
OT Site 1	GGAAAATCCACCATCCATCCTGG	chr2: 95670408-95670430	ttgaaggcaggaacttctgg	tgacagatgcaccaagtgtt
OT Site 2	GTATCATCCACAATCCATCCAAG	chr4: 111917803-111917825	agtcactgctgccaaatcct	tacacccctccccacaaata
OT Site 3	GCCAAATCCACAATCCATGCCAG	chr14: 5746379-5746401	ttctttatgcccctgtcctg	gctttcacatgggggtctaa
OT Site 4	CTCAGAAACACAATCCATCCCAG	chr3: 86410011-86410033	tcccattttgcagatggact	ctttcaccttggaggctgtt
OT Site 5	TGTACAGCCACAATCCATCCAAG	chr8: 44004281-44004303	gatggtgtagaacacccaactg	ccaccatagctcaccgaaat
OT Site 6	GTCCGAACCCCAATCCATCCTAG	chr2: 84878323-84878345	ctgggaagcataaagggaag	ttgcagctattttgtcccagt
OT Site 7	TGGAAATCCACAATCCATCTCAG	chr2: 216880248-216880270	caccaggatccccaagtcta	ctgcctcaaaatcccagtgt
OT Site 8	AGCAGATCCAGATTCCATCCAGG	chr4: 70983405-70983427	gccattctgggataccatgt	acctctgtgggaagcaaaga
OT Site 9	AGCATAACCAGAATCCATCCGGG	chr11: 78421166-78421188	tcctctcttgcctcccagta	cgcaggacaagtgtgtgatt
OT Site10	TGCATACCCAGAATCCATCCTAG	chr6: 129397603-129397625	cagagcaggtgtgtggaaga	cgtttaatcagcgagtgcag

b) SURVEYOR assay of Top 10 potential off-target sites

W OT1 W OT2 W OT3 W OT4 W OT5 W OT6 W OT7 W OT8 W OT9 W OT10

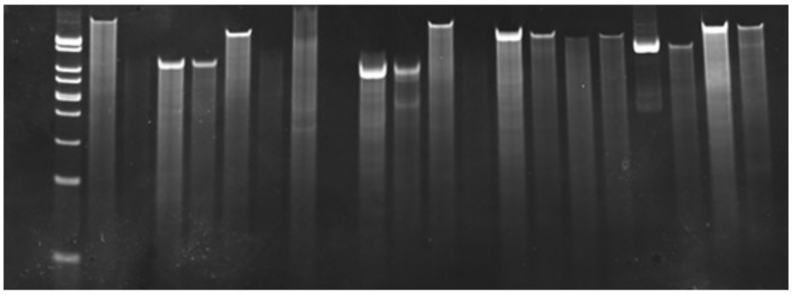

Supplementary Figure 8 - In-silico identification of the Top 10 sgRNA off-target sites and NHEJ activity assessed by the SURVEYOR assay. **a)** The top 10 OT sites were computed by taking into account the following i) total number of mismatches (either 3-4 mismatches, highlighted in red), ii) mismatch absolute position (to accommodate for the relatively high disturbance of mismatches falling close to the PAM site) and iii) mean pairwise distance between mismatches to account for the steric effect of closely neighbouring mismatches in disrupting sgRNA -DNA interaction. A total of 156 off-target sites were found, although only 14 of which were intragenic. Corresponding PCR primer pairs were designed to flank the top 10 potential off target sites. **b)** Using extracted gDNA from the founder animal and wild-type controls, gDNA products were made to flank each potential OT site (~300-500bp amplicon) and run on the SURVEYOR assay. None of the 10 OT sites tested revealed NHEJ activity and was later corroborated with sequencing data.

5. Breeding, re-derivation and maintenance at Charles River, UK

The F1 founder female was mated with male wild types at Horizon Discovery and generated F2 progeny containing the mutation confirming germ-line transmission. A total of five male positives were exported to Charles River, Lyon, France for re-derivation by embryo transfer. The resulting specific pathogen free (SPF) progeny were sent to Charles River, Margate, UK

for routine breeding and generation of experimental groups for phenotypic testing. The standard breeding protocol was a heterozygous x wild-type cross giving rise to 1:1 average *Cyfip1*$^{+/-}$ /wild-type progeny allowing full use of the litter and the generation of litter-mate controls. In most cases and unless otherwise stated, the genetic modification was transmitted through the male, resulting in the litters being raised by wild-type dams. On occasion, transmission was through the female, this was to check for parent-of-origin effects; no POE have been found. The *Cyfip1*$^{+/-}$ rat model is viable and Charles River has reported no adverse effects on breeding performance, development, general health; and in addition, no deviation from the expected Medelian 1:1 ratio of *Cyfip1*$^{+/-}$ to wild-types and no skewing of the sex ratios.

6. Housing of experimental groups at Cardiff

All experiments were carried out using adult male littermate Long Evans rats (4.5 - 9months old) that had been produced at Charles River, UK and delivered to Cardiff at 8-10 weeks of age. Rats were housed in groups of mixed wild-type/*Cyfip1*$^{+/-}$ in conventional, environmentally enriched cages, maximum 4 to a cage, with unrestricted food and water access and a standard 12h light-dark cycle (08:00 to 20:00). Room temperature was maintained at 21 ± 2°C and humidity at 55 ± 10%. Animal husbandry was carried out by technical staff at Cardiff University. All procedures were performed in accordance with ARRIVE guidelines, the local regulations set by Cardiff University and the Animal (Scientific Procedures) Act 1986.

7. PCR Genotyping

Genotyping was carried out twice, an initial assay from tissue sent from Charles River prior to transport of the rats to Cardiff and then a further confirmatory assay post-mortem, in both cases the genotyping was carried out by the investigators in Cardiff. DNA was extracted using Qiagen DNEasy Blood & Tissue Kit, Cat No./ID: 69506 and PCR carried out using a 2-primer design with primer sequences: **Forward** - TAGGGCTGCTCAGTTTTTACG, **Reverse** - TTGTTGTGGTTGG CCAGGAA. For the cycling conditions 2 ul of sample was added to 23 ul of PCR Mastermix solution (12.5 ul Promega GoTaq® G2 Green Mastermix M7822, M7823, 1 ul of each primer and 8.5 ul nuclease-free water) and placed in a thermal cycler under the following conditions: 1.) 95 °C for 10:00, 2.) 95 °C for 00:40, 3.) 60C for 00:40, 4.) 72 °C for 01:00, 5.) 40 cycles of steps 2-4, 6.) 72 °C for 05:00 and 7.) 8 °C for ever. The resulting PCR products for the mutant and wild-type sequences differed by 8bp, which produces a single

band (wild-type) or a double band (*Cyfip1*$^{+/-}$), Supplementary Figure 9. To achieve this level of resolution, 10 ul PCR product and 3ul Promega 6X Blue/Orange Loading Dye (G1881) were pipetted into a well in a 4% agarose gel and run for 1.5-2 hours at 150v.

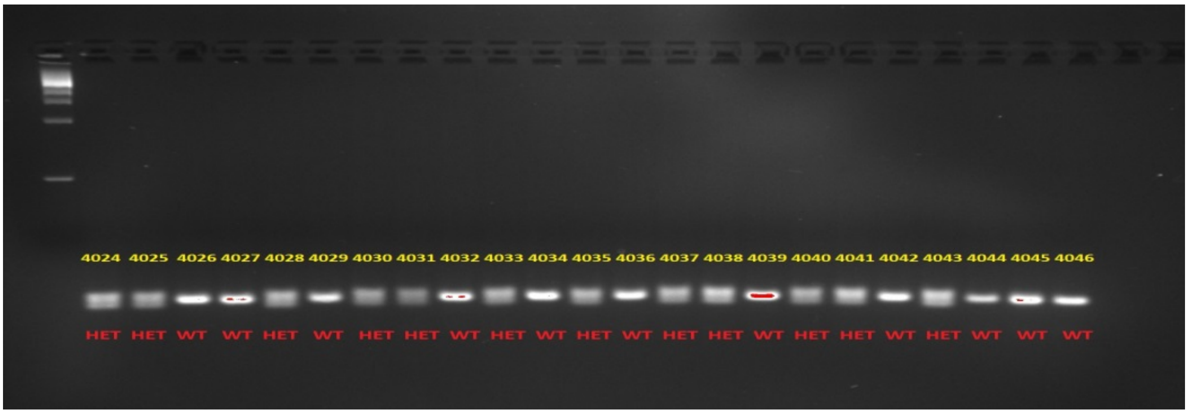

Supplementary Figure 9 - Gel image showing PCR products from *Cyfip1*$^{+/-}$ and wild-type rats using ear tissue; wild–type products show as a single band, *Cyfip1*$^{+/-}$ products as a double band, using this 2-primer design there was 100% concordance between the initial and post mortem genotype determinations.

8. Molecular verification and characterisation of the *Cyfip1* KO rat model

8.1. qRT-PCR

The effect of the heterozygous genomic heterozygous premature stop codon on transcription was tested by measuring *Cyfip1* mRNA gene expression in the brain tissue of *Cyfip1*$^{+/-}$ rats via quantitative real-time PCR. Rats were sacrificed by carbon dioxide inhalation and whole brains from mutant adult males were extracted, alongside wild-type littermate controls (5.5 - 8months old, n=11 per genotype) and hemi-brain dissected and stored on dry ice before storage at -80 °C. In a separate group of animals (4.5 - 5 months old, n=10 per genotype) prefrontal cortex (PFC) and hippocampal regions from both brain hemispheres were dissected free-hand and frozen on dry ice before storage at -80 °C. RNA was extracted from the hippocampus and PFC (one hemisphere only), and hemi-brains, using RNeasy Kits (Qiagen), followed by DNase treatment of RNA (TURBO DNA-free Kit, Ambion, Life Technologies) and cDNA synthesis (RNA to cDNA EcoDry Premix, Random Hexamers, Clontech). cDNA samples were prepared in triplicate in 96-well reaction plates for SYBR-green-based quantitative real-time PCR

(SensiFAST, HI-ROX, Bioline), according to manufacturer's instructions using a StepOnePlus System (Applied Biosystems, Thermo Fisher Scientific). *Cyfip1*-specific primers, along with *Gapdh* and *Hprt* primers (housekeeping genes), were bioinformatically designed and assessed to span at least one exon-exon boundary and to match only for its target mRNA sequence in rat (primer-BLAST and nBLAST, NCBI), before being commercially synthesised (Sigma-Aldrich). Primer efficiencies were experimentally determined through a dilution series of brain region-specific wildtype cDNA (efficiency of 90-110% was required, annealing temperature set at 60 °C). All samples were run in triplicate and individual -ΔCt values (relative to Gapdh and Hprt) were used to quantify mRNA gene expression. Primers used for qRT-PCR were as follows: *Cyfip1* (FOR: GAGGAGAATAAGTCCCGGTGG, REV: GTAGCGTGCCAGCTCAGAAA; targeting exon 11-12); *Gapdh* (FOR: TCTCTGCTCCTCCCTGTTCT, REV: TACGGCCAAATCCGTTCACA); *Hprt* (FOR: TCCTCCTCAGACCGCTTTTC, REV: ATCACTAATCACGACGCTGGG). As illustrated in Supplementary Figure 10 quantification by qRT-PCR confirmed that the genomic disruption of *Cyfip1* leads to reduced Cyfip1 mRNA expression in *Cyfip1*$^{+/-}$ rats, with a reduction seen in the hemi-brain, hippocampus and PFC compared to wild-types (HEMI-BRAIN GENE ($F_{(1,16)}$=63.094, p=0.0001), HIPP GENE ($F_{(1,18)}$=27.192, p=0.0001), PFC GENE ($F_{(1,18)}$=39.831, p=0.0001); 1-way ANOVA on -ΔCt data). We also confirmed that *Cyfip1* haploinsufficiency did not change *Cyfip2* expression (data not shown).

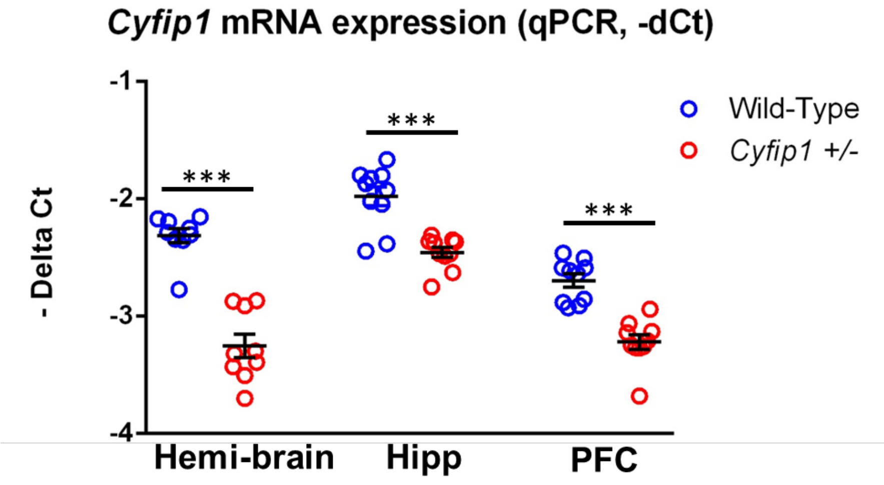

Supplementary Figure 10 - Cyfip1 mRNA expression in the hemi-brain, hippocampus and PFC and of wild-type and *Cyfip1*$^{+/-}$ rats. Measurement of –ΔCt by qRT-PCR revealed reductions of Cyfip1 mRNA in the hemi-brain of *Cyfip1*$^{+/-}$ rats compared with wild-types

(wild-type: -2.312 ± 0.187, *Cyfip1*$^{+/-}$: -3.253 ± 0.302; equivalent to a 47.9% decrease using $2^{-\Delta\Delta Ct}$). Similarly, Cyfip1 mRNA expression in *Cyfip1*$^{+/-}$ rats was reduced compared with wild-types in the hippocampus (wild-type: -1.976 ± 0.256, *Cyfip1*$^{+/-}$: -2.455 ± 0.139; equivalent to a 28.3% decrease) and PFC (wild-type: -2.695 ± 0.178, *Cyfip1*$^{+/-}$: -3.219 ± 0.193; 30.5% reduction). Four hemi-brains were of insufficient RNA quality to perform qRT-PCR (n=2 per genotype). Individual –ΔCt data is graphed with error bars representing SEM. 1-way ANOVA performed on individual data points, with normality assessed by Shapiro-Wilk tests. * = P<0.05, ** = P<0.01, *** = P<0.001. Source data are provided as a Source Data file.

8.2. Immunofluorescent western blotting

Semi-quantitative immunofluorescent western blotting assays were employed to measure Cyfip1 protein in the brain of *Cyfip1*$^{+/-}$ rats and thereby determine whether the disruption already observed at the genomic (see section 3 and 7) and mRNA expression level (see section 8.1) was maintained at the level of Cyfip1 protein. The remaining, contralaterally dissected hippocampus and PFC (see section 8.1, n=10 per genotype), hemi-brains from a separate cohort of rats (n=11 per genotype, 5.5m - 8m old), were homogenized manually with a glass Dounce homogeniser in 1 ml of ice-cold lysis RIPA buffer (RIPA Lysis and Extraction Buffer, ThermoFisher Scientific) containing protease inhibitors (cOmplete Mini EDTA-free Protease Inhibitor, Roche, 1 tablet/ 10 ml RIPA). The homogenates were centrifuged at 12,000 rpm for 20 minutes at 4°C and aliquots of supernatant containing proteins stored at -80°C. A total of 40 µg of protein (quantified using Pierce BCA Protein Kit Assay, as per manufacturer's instructions, ThermoFisher Scientific) in Laemmli sample buffer containing 1/20 β-mercaptoethanol (Bio-rad) were denatured at 95°C for 5 min prior to separation on a 4-12% gradient Bis-Tris Midi gel (NuPAGE, ThermoFisher Scientific) in 1x Bolt MES SDS Running Buffer (ThermoFisher Scientific) at a constant voltage of 115 V for 1 hr. Transfer was performed in 1 x Bolt Transfer Buffer (ThermoFisher Scientific) to Amersham Protran nitrocellulose membranes (GE Healthcare Life Sciences) at a constant voltage of 85 V for 2 hr 15 min at 4 °C. Blots were blocked in 5% non-fat milk (Blocking Agent, Amersham, GE Healthcare Life Sciences) in 0.01 M Tris buffered saline solution containing 0.2% Tween 20 (TBST), and this TBST solution was used for all subsequent washes. Primary and fluorescent secondary antibodies were similarly diluted in TBST containing 0.2% Tween 20 and 5% milk and they were used at the following concentrations: Cyfip1 (AB6046, Millipore), 1:1,000;

Gapdh (ab8245, Abcam), 1:5,000; IRDye® 680RD Goat anti-Rabbit IgG (Li-Cor, 926-68071), 1:15,000 and IRDye® 680RD Donkey anti-Mouse IgG (Li-Cor, 926-68072), 1:15,000. Incubation of blots in primary antibody solutions were at 4°C overnight, whilst fluorescent secondary antibodies were for 1 hr at RT. Blots were visualised using the 700 nm channel of the Odyssey CLx Imaging System (Li-Cor) and densitometric quantification was performed on scanned blot films using ImageStudio Lite software (Li-Cor). Densitometry data was normalized to the protein loading control (Gapdh) and between-blot variance was minimised by normalising each *Cyfip1*$^{+/-}$ sample signal to the average signal of all the wildtype samples on the same blot.

Supplementary Figure 11 shows an overall 33% reduction in Cyfip1 protein levels in the hemi-brain samples of *Cyfip1*$^{+/-}$ compared with wild-types (Hemi-brain Cyfip1 prot ($F_{(1,20)}=6.618$, $p=0.018$); 1-way ANOVA). Similar levels of mean reductions were found in the hippocampal and prefrontal cortex samples. However, due to a greater degree of variability the similar effect size to the hemi-brain samples did not result in a significant difference between groups (Hipp Cyfip1 prot, ($F_{(1,18)}=2.503$, $p=0.131$), 1-way ANOVA; PFC Cyfip1 prot ($F_{(1,20)}=1.811$, $p=0.195$), 1-way ANOVA). The increased variability in the hippocampal and prefrontal cortex samples was common to both the wild-type and *Cyfip1*$^{+/-}$ rats, so it is unlikely to be due to the genetic manipulation *per se*. Whether these data reflect a degree of variability in the extent to which the reductions in mRNA translate into systematic reductions in protein levels, at least in the hippocampus and PFC, remains to be determined.

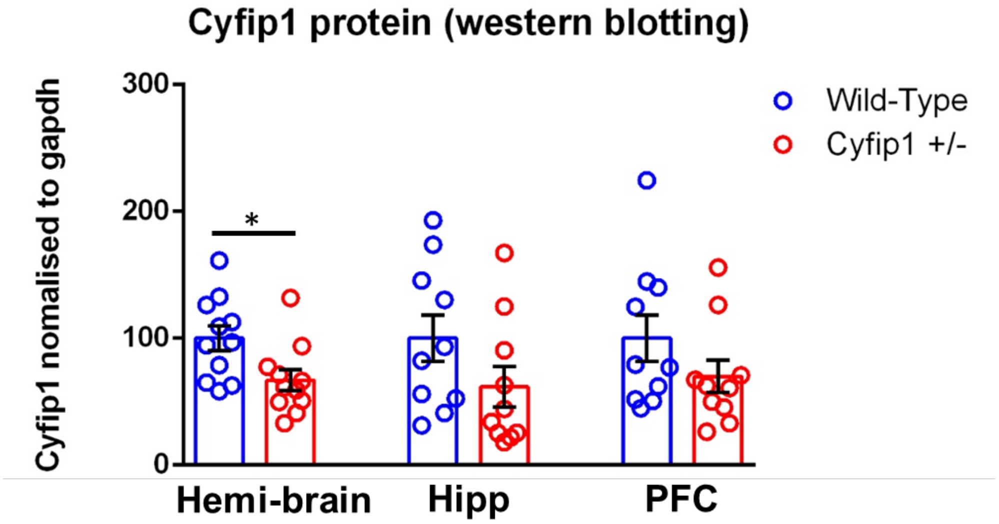

Supplementary Figure 11 - Cyfip1 protein levels in the hemi-brain, hippocampus and PFC of wild-type and *Cyfip1*$^{+/-}$ rats. Measurement of protein levels by immunofluorescent western blotting revealed reductions of Cyfip1 protein in the hemi-brain of *Cyfip1*$^{+/-}$ rats compared with wild-types (wild-type: 100.0 ± 9.8%, *Cyfip1*$^{+/-}$: 67.0 ± 8.3%; 33.0% decrease). However, Cyfip1 protein in the hippocampus and PFC of *Cyfip1*$^{+/-}$ rats was not significantly reduced (Hippocampus wild-type: 100.0 ± 18.2%, *Cyfip1*$^{+/-}$: 61.6 ± 16.1%; PFC wild-type: 100.0 ± 18.2%, *Cyfip1*$^{+/-}$: 70.0 ± 12.9%). Densitometric data given relative to the average of all wild-type samples (100%) and normalised to protein loading control, Gapdh. Bars represent SEM, and 1-way ANOVA performed on individual data points. * = $P<0.05$, ** = $P<0.01$, *** = $P<0.001$. Source data are provided as a Source Data file.

Supplementary References

1. Anderson, K. R. *et al.* CRISPR off-target analysis in genetically engineered rats and mice. *Nat. Methods* **15**, 512–514 (2018).
2. Hsu, P. D. *et al.* DNA targeting specificity of RNA-guided Cas9 nucleases. *Nat. Biotechnol.* **31**, 827–832 (2013).
