# Supplementary material for: Cyfip1 haploinsufficient rats show white matter changes, myelin thinning, abnormal oligodendrocytes and behavioural inflexibility
Source: Nat Commun. 2019 Aug 1;10:3455. doi: 10.1038/s41467-019-11119-7 (PMC6671959; doi:10.1038/s41467-019-11119-7)
Supplement: Supplementary file 3 — Reporting Summary [file 41467_2019_11119_MOESM3_ESM.pdf]

nature research

Corresponding author(s): Lawrence Wilkinson

Last updated by author(s): Jun 6, 2019

Reporting Summary

Nature Research wishes to improve the reproducibility of the work that we publish. This form provides structure for consistency and transparency in reporting. For further information on Nature Research policies, see Authors & Referees and the Editorial Policy Checklist.

Statistics

For all statistical analyses, confirm that the following items are present in the figure legend, table legend, main text, or Methods section.

n/a	Confirmed	
☐	☒	The exact sample size (*n*) for each experimental group/condition, given as a discrete number and unit of measurement
☐	☒	A statement on whether measurements were taken from distinct samples or whether the same sample was measured repeatedly
☐	☒	The statistical test(s) used AND whether they are one- or two-sided<br>*Only common tests should be described solely by name; describe more complex techniques in the Methods section.*
☐	☒	A description of all covariates tested
☐	☒	A description of any assumptions or corrections, such as tests of normality and adjustment for multiple comparisons
☐	☒	A full description of the statistical parameters including central tendency (e.g. means) or other basic estimates (e.g. regression coefficient) AND variation (e.g. standard deviation) or associated estimates of uncertainty (e.g. confidence intervals)
☐	☒	For null hypothesis testing, the test statistic (e.g. *F*, *t*, *r*) with confidence intervals, effect sizes, degrees of freedom and *P* value noted<br>*Give P values as exact values whenever suitable.*
☒	☐	For Bayesian analysis, information on the choice of priors and Markov chain Monte Carlo settings
☒	☐	For hierarchical and complex designs, identification of the appropriate level for tests and full reporting of outcomes
☒	☐	Estimates of effect sizes (e.g. Cohen's *d*, Pearson's *r*), indicating how they were calculated

Our web collection on statistics for biologists contains articles on many of the points above.

Software and code

Policy information about availability of computer code

Data collection	MRI scanner (Bruker, Karlsruhe, Germany)
Data analysis	ExploreDTI 4.8.3 and SPM (version 12, UCL, London, UK) were used for preprocessing of the DTI data, and FSL was used for whole-brain statistical analyses. All other analyses and plotting was performed in RStudio statistical software version 1.1.463 (R Foundation for Statistical Computing, Vienna, Austria). ImageJ software (version 1.51) was used for cell quantification.

For manuscripts utilizing custom algorithms or software that are central to the research but not yet described in published literature, software must be made available to editors/reviewers. We strongly encourage code deposition in a community repository (e.g. GitHub). See the Nature Research guidelines for submitting code & software for further information.

Data

Policy information about availability of data

All manuscripts must include a data availability statement. This statement should provide the following information, where applicable:

- Accession codes, unique identifiers, or web links for publicly available datasets
- A list of figures that have associated raw data
- A description of any restrictions on data availability

All data from this study are available from the corresponding author upon reasonable request.

Field-specific reporting

Please select the one below that is the best fit for your research. If you are not sure, read the appropriate sections before making your selection.

☒ Life sciences ☐ Behavioural & social sciences ☐ Ecological, evolutionary & environmental sciences

For a reference copy of the document with all sections, see nature.com/documents/nr-reporting-summary-flat.pdf

Life sciences study design

All studies must disclose on these points even when the disclosure is negative.

Sample size	A cohort of 24 rats (12 per genotype) were used for diffusion tensor imaging, a number used typically in rodent imaging studies, and with sufficient power to detect genotype effects in this study. For electron microscopy 9 rats were used (5 WT and 4 Cyfip1 hets); in this detailed ultrastructural analyses around 13 000 axons were analysed in total as a representative sample, which is significantly in excess of published reports where similar analyses have been done. For immunofluorescence 14 rats were used (7 per genotype). Here, at least 5 random regions were quantified. For the in vitro oligodendrocyte culture experiments 3 biological repeats were used (which is the recommended standard procedure) and at least 5 images were collected from each well (8 wells per replicate), resulting in a total representative sample of ~14 000 cells. For the reversal learning work we used 7 WT and 9 hets on the basis of published reports using touch-screen methods in rats and our own experience with the paradigm. There was less previous information on the mismatch task so we increased the n accordingly, WT 21/hets 15.
Data exclusions	No data were excluded
Replication	In all the experiments each animal was a biological repeat. The oligodendrocyte culture experiments also utilised a triple technical repeat design. In both the electron microscope and cell culture experiments the representative samples were obtained from very large numbers of determinations (for the EM over 13,000 separate determinations, for the culture work 14,000 in total including 1688 cells analysed for the MBP area data). We did not perform additional repeats of the DTI study. For the behaviour we did not repeat the study for the individual paradigms but we did however, as noted in the manuscript, obtain converging evidence from both assays of effects on psychological processes underlying behavioural flexibility.
Randomization	All allocations were random.
Blinding	The investigator was blind to the genotype in all analyses done.

Reporting for specific materials, systems and methods

We require information from authors about some types of materials, experimental systems and methods used in many studies. Here, indicate whether each material, system or method listed is relevant to your study. If you are not sure if a list item applies to your research, read the appropriate section before selecting a response.

Materials & experimental systems

n/a	Involved in the study	
☐	☒	Antibodies
☒	☐	Eukaryotic cell lines
☒	☐	Palaeontology
☐	☒	Animals and other organisms
☒	☐	Human research participants
☒	☐	Clinical data

Methods

n/a	Involved in the study	
☒	☐	ChIP-seq
☒	☐	Flow cytometry
☐	☒	MRI-based neuroimaging

Antibodies

Antibodies used	anti-Olig2 (ab109186, Abcam), anti-APC [CC-1] (ab16794, Abcam) , anti-MBP (MAB386, Millipore) , anti-O4 (MAB345, Millipore)
Validation	anti-Olig2 (ab109186, Abcam): host species rabbit, and shown to react with mouse, rat and human. This product has been referenced at least 23 times. More info can be found in: https://www.abcam.com/olig2-antibody-epr2673-ab109186.html?productWallTab=ShowAll#top-255<br><br>anti-APC [CC-1] (ab16794, Abcam): host species mouse, and shown to react with rat, human and monkey. It has been referenced at least 40 times. More info can be found in: https://www.abcam.com/apc-antibody-cc-1-ab16794.html?productWallTab=ShowAll<br><br>anti-MBP (MAB386, Millipore): host species rat, reacts with MBP from all species tested including human, bovine, sheep, rabbit, mouse, rat, guinea pig and chicken. It has been references multiple times and all the supporting documentation can be found in

the website: http://www.merckmillipore.com/GB/en/product/Anti-Myelin-Basic-Protein-Antibody-a.a.-82-87,MM_NF-MAB386

anti-O4 (MAB345, Millipore): host species mouse, reacts with chicken, human, mouse, and rat. It has been referenced multiple times and supporting documentation can be found in the website: http://www.merckmillipore.com/GB/en/product/Anti-O4-Antibody-clone-81,MM_NF-MAB345?ReferrerURL=https%3A%2F%2Fwww.google.com%2F.

Animals and other organisms

Policy information about studies involving animals; ARRIVE guidelines recommended for reporting animal research

Laboratory animals	All the rats used in this study were Long Evans males. The age of the rats slightly differed in each experiment: the rats used in DTI were 5 months old and were euthanized 1 month after the scanning for immunofluorescence. The rats used for electron microscopy were 6 months old, and the rats used for behavioural experiments were between 6 to 9 months old.
Wild animals	NA
Field-collected samples	NA
Ethics oversight	All the experimental procedures were performed in accordance with institutional animal welfare and ARRIVE guidelines and the UK Home Office License PPL 30/3135.

Note that full information on the approval of the study protocol must also be provided in the manuscript.

Magnetic resonance imaging

Experimental design

Design type	NA
Design specifications	NA
Behavioral performance measures	NA

Acquisition

Imaging type(s)	Diffusion Tensor Imaging
Field strength	9.4 T
Sequence & imaging parameters	The MRI protocol included DTI acquisition with a diffusion-weighted (DW) spin-echo echo-planar-imaging (EPI) pulse sequence. T2 weighted images were also acquired for anatomical reference. For DTI acquisition two EPI segments were applied with gradient strength up to 600 mT/m and duration of 4.5 ms and separation of 10.5 ms, with 60 noncollinear gradient directions with a single b-value shell at 1000 s/mm^2 and one image with a b-value of 0 s/mm^2. TR = 4000 ms and a TE = 22 ms. Geometrical parameters were: 34 slices, each 0.32 mm thick (brain volume) and with in-plane resolution of 0.32x0.32 mm^2 (matrix size 80x96; FOV 25.6x30.73 mm^2). For the T2 weighted images acquisition a multi-slice multi-echo pulse sequence was used with the following parameters: TR = 7200ms, TE = 15ms and effective TE of 45ms, rare factor was 8. Image resolution was set to 0.22 mm^3 with matrix size of 128x160x50 to cover the entire brain.
Area of acquisition	whole brain

Diffusion MRI ☒ Used ☐ Not used

Parameters: 60 noncollinear gradient directions with a single b-value shell at 1000 s/mm^2 and one image with a b-value of 0 s/mm^2.

Preprocessing

Preprocessing software	ExploreDTI 4.8.371 and SPM (version 12, UCL, London, UK) were used in the preprocessing of the rat DTI data. Brain was extracted following the protocol described in the paper and using mainly SPM and ExploreDTI masking option.
Normalization	The data was normalized using the non linear transformation described in TBSS protocol (https://fsl.fmrib.ox.ac.uk/fsl/fslwiki/TBSS/UserGuide).
Normalization template	We used a population-specific template.
Noise and artifact removal	Eddy-current induced distortion and motion correction were performed, and data was also corrected for field inhomogeneities using ExploreDTI 4.8.371 (the protocol is described in the manuscript). The data was visually inspected and data quality was performed using ExploreDTI 4.8.371.
Volume censoring	NA

Statistical modeling & inference

Model type and settings	Voxel-wise independent t-tests
Effect(s) tested	Genotypic effect was studied, where 2 groups were compared.

Specify type of analysis: ☐ Whole brain ☐ ROI-based ☒ Both

Anatomical location(s)	regions of interest were defined having into account the regions showing significant differences in the whole brain analyses.
Statistic type for inference (See Eklund et al. 2016)	Using the randomize function (part of FSL), the null distribution was built over 1000 random permutations, using the Threshold-Free Cluster Enhancement (TFCE) algorithm where cluster-like structures are enhanced, and the results are shown for p<0.05.
Correction	FWE and FDR corrections were used

Models & analysis

n/a	Involved in the study
☒	☐ Functional and/or effective connectivity
☒	☐ Graph analysis
☒	☐ Multivariate modeling or predictive analysis
